# Bioavailability of oxycodone after administration of a new prolonged-release once-daily tablet formulation in healthy subjects, in comparison to an established twice-daily tablet

**DOI:** 10.5414/CP203005

**Published:** 2017-09-21

**Authors:** Bernhard Scheidel, Martina A. Maritz, Yves J. Gschwind, Kerstin Steigerwald, Volker Guth, Peter Kovacs, Helene Rey

**Affiliations:** 1 ACC GmbH, Analytical Clinical Concepts, Leidersbach, Germany,; 2 Develco Pharma Schweiz AG, Pratteln, Switzerland, and; 3 Clinical Pharmacology Unit, Institute for Internal Medicine, University of Debrecen, Debrecen, Hungary

**Keywords:** oxycodone, bioavailability, pharmacokinetics, once daily, prolonged release

## Abstract

Objective: To evaluate and to compare the bioavailability, the influence of food intake on the bioavailability, and the safety and tolerability of a newly-developed oxycodone once-daily (OOD) prolonged-release tablet with an established oxycodone twice-daily (OTD) prolonged-release tablet after single-dose administration under fasting or fed conditions as well as after multiple-dose administration. Materials and methods: Three single-center, open-label, randomized, balanced, two-treatment, two-period, two-sequence crossover studies were conducted. In each study, 36 healthy volunteers were randomized to receive 10 mg oxycodone daily as OOD (oxycodone HCL 10-mg PR tablets XL (Develco Pharma Schweiz AG, Pratteln, Switzerland); administration of 1 tablet in the morning) or as OTD (reference formulation: oxygesic 5-mg tablets (Mundipharma GmbH, Limburg an der Lahn, Germany); administration of 1 tablet in the morning and 1 tablet in the evening). Tablets were administered once daily or twice daily under fasting conditions (study 1) or under fed conditions (study 2) as well as after multiple-dose administration (study 3). A sufficient number of blood samples were taken for describing plasma profiles and for calculation of pharmacokinetic parameters. Plasma concentrations of oxycodone were determined by LC-MS/MS. Safety and tolerability were monitored and assessed in all three studies. Results: Plasma profiles of OOD reveal sustained concentrations of oxycodone over the complete dosing interval of 24 hours. In comparison to the OTD reference formulation, the OOD test formulation showed a slightly slower increase of concentrations within the absorption phase and similar plasma concentrations at the maximum and at the end of the dosing interval (24 hours). Extent of bioavailability (AUC), maximum plasma concentrations (C_max_), and plasma concentrations at the end of the dosing interval (C_τ,ss,24h_) of OOD could be classified as comparable to OTD considering 90% confidence intervals (CIs) and acceptance limits of 80.00 – 125.00%. Bioavailability of OOD was not influenced by concomitant food intake. OOD and OTD were generally well tolerated, a difference between the two products could not be observed. Conclusion: The new 10-mg OOD formulation provides sustained oxycodone plasma concentrations over the dosing interval of 24 hours and is suitable for once-daily administration. Bioavailability of OOD could be classified as comparable to the twice-daily administration of the OTD reference formulation. The new formulation widens and optimizes the range of strong opioid drug products in patient-centered therapy of chronic pain with simplified dosing and better compliance.

## Introduction 

Oxycodone is a pure opioid agonist with affinity for κ-, µ-, and δ-opiate receptors in the brain, spinal cord, and peripheral organs [1, 2]. 

The principle action of oxycodone is analgesia. Oxycodone is a strong opioid according to the World Health Organisation analgesic ladder for treating cancer pain (same as morphine, hydromorphone, fentanyl, and methadone) [3, 4]. Oxycodone has been in clinical use since 1917 and is generally indicated for the treatment of severe chronic pain requiring the use of a strong opioid, although indications vary between countries [1, 2, 5]. Although morphine has been the standard opioid analgesic in the management of moderate-to-severe pain for many years, according to opioid consumption across the world, oxycodone is the most widely used in the treatment of severe pain [5]. Oxycodone is available in a range of formulations that include intravenous ampoules for injection or infusion, immediate-release solution, capsules for oral administration every 4 – 6 hours (all traded as OxyNorm/Oxygesic; Napp/Mundipharma) [6], and prolonged-release tablets for oral administration every 12 hours (traded as OxyContin/Oxygesic; Napp/Mundipharma) [7, 8]. 

Results of a recent survey on patients’ and physicians’ perspectives on opioid therapy of pain suggests that a lower number of intakes per day correspond to higher satisfaction and convenience for chronic pain patients [9]. A finding that supports the association between simpler, less frequent treatment regimens and better patient compliance [10]. 

A new oxycodone prolonged-release tablet formulation was developed to allow for administration every 24 hours. The aim was to design a formulation with even more extended prolonged-release characteristics allowing for simplified once-daily dosing regimen while attaining stable diurnal plasma concentrations and minimizing fluctuations in exposure over time, especially the 12-hour trough concentrations associated with the twice-daily administration of established prolonged-release oxycodone formulation. The studies presented here investigate bioavailability, influence of food on bioavailability as well as safety and tolerability of this newly-developed oxycodone once-daily (OOD) formulation in healthy subjects in comparison to the established oxycodone twice-daily (OTD) product. 

## Materials and methods 

### Study design and formulations 

Three studies were conducted as single-center, open-label, randomized, balanced, two-treatment, two-period, two-sequence crossover studies. Washout periods of at least 7 days separated the two treatment periods in each study. The first study considered a once-daily administration of OOD and a twice-daily administration of OTD under fasting conditions (study 1, EudraCT-No.: 2008-008216-19) and the second study considered these under fed conditions (study 2, EudraCT-No.: 2009-012646-24). The third study included multiple-dose administration of OOD and OTD (study 3, EudraCT-No.: 2009-012648-17). 

Products under investigation in all three studies were the 10-mg strength of a new OOD tablet formulation as test product (named Oxycodone HCl 10-mg PR tablets XL (Develco Pharma Schweiz AG)) and the 5-mg strength of an established OTD tablet formulation as reference product (traded Oxygesic 5-mg Retardtabletten, Mundipharma GmbH, Limburg an der Lahn, Germany). 

The test product is a prolonged-release multiple-unit pellet formulation intended for administration every 24 hours. Available tablet strengths of 10 mg, 20 mg, 40 mg, and 80 mg showed dose proportionality [11]. The new formulation is designed to also have abuse-deterrent properties regarding expected methods of abuse (crushing, heating, snorting and intravenous application) and to also have alcohol-resistant properties regarding misuse by concomitant alcohol consumption [12]. 

The reference product is a matrix formulation where the release of oxycodone is biphasic with an initial relatively fast release followed by a more controlled release [7, 8, 13]. 

The reference product is approved for administration every 12 hours and is labelled as a prolonged-release tablet, but is also referred to as controlled release [13, 14, 15]. 

### Subjects 

In each of the three studies, 36 healthy male and female Caucasian subjects between 18 and 55 years of age with a BMI of 19 – 28 kg/m^2^ were enrolled. Female subjects had to use contraception and undergo pregnancy tests before and during the study. Assessment of subjects’ health was based on medical and medication history, physical examinations, 12-lead electrocardiogram, and clinical laboratory testing. Main exclusion criteria were smoking (> 10 cigarettes per day), intake of any medication (prescribed or over-the-counter, including herbal remedies) up to 2 weeks before first study medication administration, history of compulsive alcohol (≥ 10 drinks weekly) and/or regular drug abuse, and any health conditions where opioids are contraindicated. Written informed consent was obtained from all subjects prior to inclusion. The studies were approved by the Hungarian Medical Research Council Ethics Committee for Clinical Pharmacology and were performed in accordance with regulatory and legal requirements, Good Clinical Practice guidelines [16], and the Declaration of Helsinki [17]. 

### Study procedures 

In each treatment period of study 1 and study 2, subjects were required to stay at the clinical unit for at least 12 hours before the morning administration and were discharged 24 hours thereafter. Subjects returned to the clinical unit for blood sampling 30, 36, and 48 hours after the morning administration. In each treatment period of study 3, subjects were required to begin their stay at the clinical unit at least 12 hours before first administration, ending their stay in the morning of day 5 (i.e., 12 hours after last administration). On blood sampling days, the subjects swallowed the medication in a seated position and then stayed in a semireclined position until 3 hours post-dose. 

Study 1 (fasting conditions): At 07:00 AM, after at least a 10-hour overnight fast, the subjects received either 1 tablet of OOD 10 mg or the first tablet of OTD 5 mg. Twelve hours after the first dose, the second tablet of OTD 5 mg was administered. A standardized light breakfast and a cooked lunch were served 3 hours and 6 hours after morning dose, respectively. Dinner was served 15 hours after morning dose. 

Study 2 (fed conditions): Between 06:30 and 06:50 AM, after an overnight fast of at least 10 hours, the subjects consumed a standard high-fat, high-calorie breakfast as recommended by the US Food and Drug Administration [18]. Afterwards, either OOD 10 mg or the first tablet of OTD 5 mg was administered. Twelve hours after the first dose, the second tablet of OTD 5 mg was administered. A cooked lunch was served 6 hours and a dinner 15 hours after morning dose. 

Study 3 (multiple-dose administration): On 4 consecutive days at 07:00 AM and after at least a 10-hour overnight fast subjects received either OOD 10 mg or the first tablet of OTD 5 mg. Twelve hours after the first dose, the second tablet of OTD 5 mg was administered. Standard meals were consumed 3, 6, and 15 hours after morning dose. 

### Blood sampling 

To determine blood plasma concentrations of oxycodone in study 1 and study 2, blood samples were collected at 0 (pre-dose), 0.5, 1.0, 1.5, 2.0, 2.5, 3.0, 3.5, 4.0, 4.5, 5.0, 6.0, 7.0, 8.0, 10.0, 12.0, 14.0, 16.0, 20.0, 24.0, 30.0, 36.0, and 48.0 hours post OOD administration and at 0 (pre-dose), 0.5, 1.0, 1.5, 2.0, 2.5, 3.0, 3.5, 4.0, 4.5, 5.0, 6.0, 7.0, 8.0, 10.0, 12.0, 12.5, 13.0, 13.5, 14.0, 14.5, 15.0, 15.5, 16.0, 16.5, 17.0, 18.0, 19.0, 20.0, 22.0, 24.0, 30.0, 36.0, and 48.0 hours post OTD administration. More frequent samples were collected between 12 and 24 hours after the morning OTD dose to describe the profile for the evening OTD dose. 

In the multiple-dose study, study 3, pre-dose blood samples were collected on days 1 – 4, and postdose blood samples were collected on day 4 at the same time point as in studies 1 and 2, until 24 hours postdose. 

Blood samples were collected into vacutainers, immediately cooled in an ice bath, and centrifuged within 30 minutes. Plasma was transferred and stored at –20 °C until analysis. 

### Bioanalysis 

Oxycodone plasma concentrations were quantified using a validated method [19] that used a liquid-liquid extraction for sample preparation and liquid chromatography with tandem mass spectrometry detection (LC-MS/MS API 2000, PE Biosystems, Foster City, CA, USA) for sample measurement. Oxycodone was selectively quantified by using multiple-reaction monitoring as detection mode, hydromorphone was used as internal standard. The calibration curve of oxycodone ranged from 0.5 to 100.0 ng/mL. The lower limit of quantitation (LLOQ) was 0.5 ng/mL. Range of intraday precision was determined as 2.6 – 5.6% for 80.0 ng/mL, as 2.0 – 8.3% for 10.0 ng/mL, as 3.4 – 4.4% for 1.0 ng/mL, and as 3.6 – 6.9% for 0.5 ng/mL. Interday precision was 5.6% for 80.0 ng/mL, 6.5% for 10.0 ng/mL, 3.6% for 1.0 ng/mL, and 6.5% for 0.5 ng/mL. Range of intraday accuracy was determined as –0.1 to 9.4% for 80.0 ng/mL, as –6.0 to 3.0% for 10.0 ng/mL, as –2.0 to 0.0% for 1.0 ng/mL, and as 0.8 to 11.8% for 0.5 ng/mL. Interday accuracy was 5.6% for 80.0 ng/mL, –2.0% for 10.0 ng/mL, –1.0% for 1.0 ng/mL, and 6.2% for 0.5 ng/mL. 

Stability was investigated and documented for storage of stock solution (up to 11 weeks at 2 – 8 °C), for three thaw/freeze cycles, for plasma sample processing (up to 6 hours at room temperature), for autosampler waiting time (up to 24 hours at 2 – 8 °C), and for long-term storage (up to 9 months at –20°C). 

### Pharmacokinetic data evaluation 

Pharmacokinetic parameters for oxycodone were calculated by standard noncompartmental methods (WinNonlin Version 5.2.1, Pharsight Corporation, Mountain View, CA, USA). 

Pharmacokinetic parameters were chosen in order to describe and compare the bioavailability of OOD and OTD as well as to investigate the influence of food on the bioavailability of each formulation. 

As primary parameter for describing the extent of bioavailability, the area under the plasma concentration time curve (AUC) was calculated in study 1 and study 2 for OOD and OTD as AUC_(0–t)_ (from time 0 to last measured concentration (t)) and as AUC_(0–∞)_ (from time 0 to infinity), whereby for OTD the AUC considered the sum of the administrations in the morning and in the evening. Residual area (%) was calculated using the formula (AUC_(0–∞)_ – AUC_(0–t)_) × 100/AUC_(0–∞)_. Additionally, for OTD, AUC_(0–12h)_ was calculated for assessment of influence of food. In study 3, AUC was evaluated for OOD and OTD as AUC_ss(0–24h)_ (from time 0 to 24 hours) considering for OTD the sum of the two 12-hour dosing intervals. 

As secondary parameters, the maximum plasma concentration (C_max_), the corresponding time to reach maximum plasma concentration (t_max_), and the elimination half-life (T_1/2_) were evaluated in study 1 and study 2, in addition, the concentration at the end of the dosing interval (C_τ,ss,24h_), the half value duration (HVD), and the peak-trough fluctuation (PTF) were evaluated in study 3. 

C_max_ and t_max_ were evaluated in study 1 and study 2 for OOD and OTD as C_max(0–t)_ and as t_max(0–t)_. Additionally, C_max(0–12h)_ and t_max(0–12h)_ were evaluated for assessment of food effect of OTD. In study 3, C_max_ was evaluated for OOD and OTD as C_max,ss(0–24h)_. 

T_1/2_ was calculated within study 1 and study 2 by using the formula ln(2)/λ_z_ whereby the terminal rate constant λ_z_ was obtained by linear regression using log-transformed plasma concentrations of the terminal elimination phase. 

After multiple-dose administration (study 3) the concentrations at the end of the dosing intervals were evaluated for OOD and OTD as C_τ,ss,24h_. 

The half-value duration reflects the duration in which the plasma concentration is at or above 50% of maximum plasma concentration (also referred to as T50% C_max_) [20] and was calculated in study 3 for OOD and OTD as HVD_ss(0–24h)_. 

The peak-trough fluctuation was calculated by using the formula (C_max,ss(0–24h)_ – C_min,ss(0–24h)_)/C_av,ss(0–24h)_ whereby C_av,ss(0–24h)_ was obtained by the formula AUC_ss(0–24h)_/24h. 

### Statistical analysis 

Statistical analyses were performed using SAS 9.1.3 (SAS Institute Inc., Cary, NC, USA). 

To compare bioavailability of OOD and OTD, 90% confidence intervals (CIs) were calculated for AUC_(0–t)_ and C_max(0–t)_ (study 1 and study 2) and for AUC_ss(0–24h)_, C_max,ss(0–24h)_, and C_τ,ss,24h_ (study 3). Logarithmic transformations were applied for analysis of variance (ANOVA). 90% CIs for the difference between drug formulation least-squares means (LSM) were calculated. Comparability between OOD and OTD was assumed if the retransformed CIs of the different parameters were within the acceptance range of 80.00 – 125.00%. 

To assess the influence of food on bioavailability of OOD or OTD, results of study 1 (fasting conditions) and study 2 (fed conditions) were compared and statistically handled as interstudy comparison by using a parallel-group design. This procedure was justified because the population of subjects, blood sampling points, study procedures, clinical unit, and bioanalytical method were the same for both studies. AUC_(0–t)_ and C_max(0–t)_ of OOD and AUC_(0–12h)_ and C_max(0–12h)_ of OTD were chosen to compare the results obtained under fasting or fed conditions for the respective formulation. Non-zero log-transformed data were used for ANOVA. Data were subjected to a one-way ANOVA using the SAS GLM procedure for the estimation of the LSM of the factor “condition”. 90% CIs for the difference between drug formulation geometric least-squares means (LSMEANS) were calculated for the log-transformed parameters. 

### Safety assessment 

Study subjects were monitored for changes in health status and clinical parameters. Physical examination including vital signs, 12-lead electrocardiogram, and routine clinical laboratory test (i.e., hematology, clinical chemistry, serology, urinalysis) were performed at screening and at follow-up. Prior to each treatment period, tests for pregnancy, vital signs, and electrocardiogram were performed. Prior to each blood sampling (including screening and follow-up), subjects were questioned about adverse events (AEs). 

## Results 

### Demographic data of subjects 

Demographic data of the subjects of the studies are summarized in Table 1. In each study, 36 subjects were enrolled and completed the study. There were no withdrawals or drop outs. In terms of age, body mass index, weight, and height there were no obvious differences between the collectives of the three studies. 

### Comparison of bioavailability 

**Figure 1.**
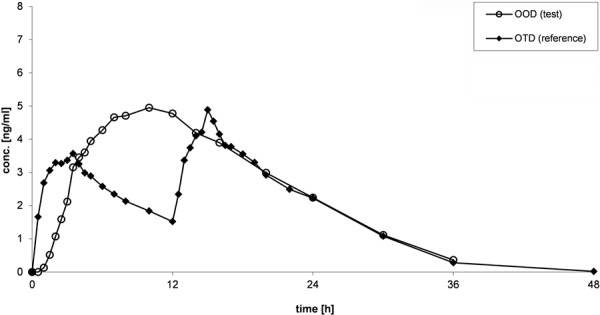
Mean (arithmetic mean) plasma concentration-time curves of oxycodone after administration of OOD and OTD under fasting conditions (study 1).

Mean plasma concentration-time curves of oxycodone after administration of OOD and OTD are presented in Figure 1 (study 1: fasting conditions), 2 (study 2: fed conditions), and Figure 3 (study 3: multiple-dose administration). 

**Figure 2:**
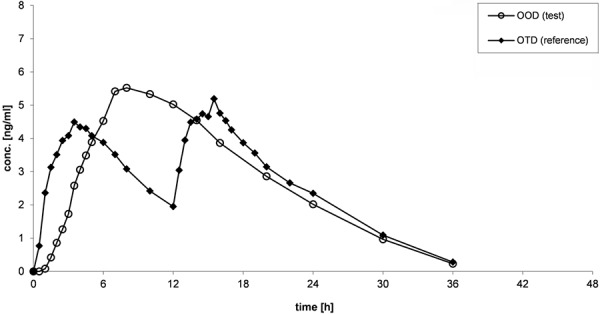
Mean (arithmetic mean) plasma concentration-time curves of oxycodone after administration of OOD and OTD under fed conditions (study 2).

For both OOD and OTD, similar shapes of curves could be observed under fasting and fed conditions (Figures 1, 2). During the absorption phase, increase of concentrations are slower for OOD than for OTD, which is characterized by a rapid absorption. Shape of curves of OOD reveal a continuous increase, and maximum plasma concentrations are reached between 8 and 12 hours after administration. Maximum plasma concentrations of OTD are reached after the morning dose as well as after the evening dose between 2 and 4 hours after administration. Within the elimination phase, the plasma profiles between OOD and OTD are similar. 

**Figure 3.**
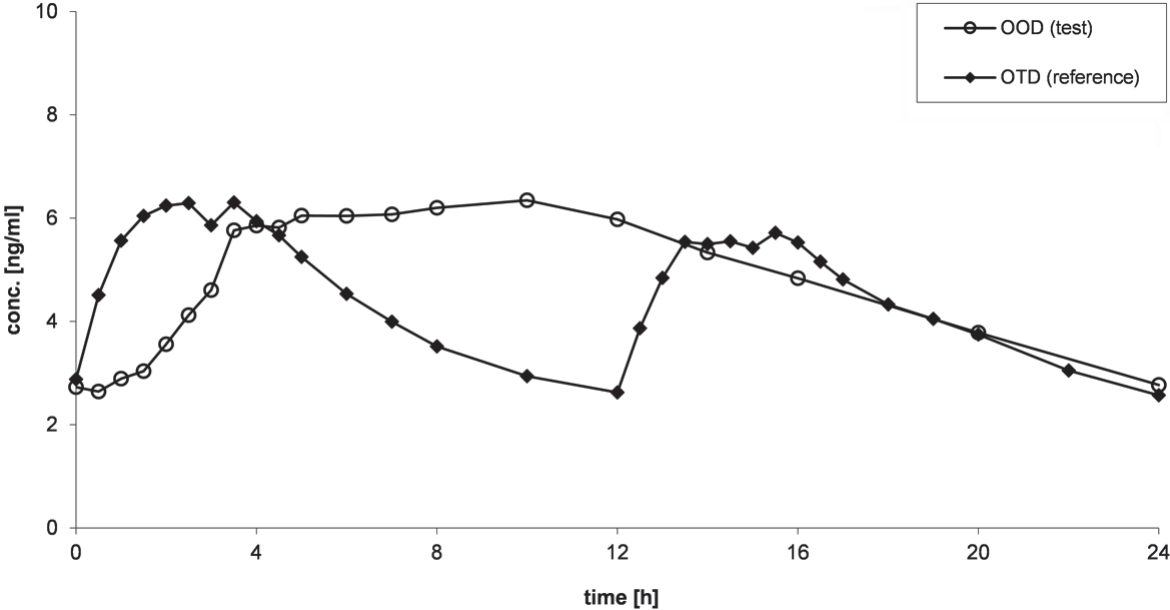
Mean (arithmetic mean) plasma concentration-time curves of oxycodone after administration of OOD and OTD after multiple-dose administration (study 3).

After multiple-dose administration of OOD and OTD, plasma concentrations of oxycodone are comparable between both formulations before administration on day 4 (Figure 3). The shapes of the plasma profiles confirmed the observations from study 1 and study 2. 

Pharmacokinetic parameters of oxycodone following administration of OOD and OTD under fasting and fed conditions are given in Table 2 and, after multiple dose administration, in Table 3. Within the single-dose studies (study 1 and study 2), blood sampling over the time period of 48 hours was sufficient, and LLOQ was low enough so that residual area was lower than 20%. Furthermore, AUC_(0–t)_ covered more than 80% of AUC_(0–∞)_ in 140 of 144 individual plasma concentration-time curves. The wash-out phase of 7 days covers more than 14 elimination half-lives and was therefore long enough to avoid predose concentrations in the second study period. 

The geometric mean ratios and 90% CIs of AUC, C_max_, and C_τ,ss,24h_ are provided in Table 4 for comparison of the bioavailability of OOD and OTD. The results demonstrate that all calculated CIs are within the acceptance range of 80.00 – 125.00%. 

### Influence of food on bioavailability 

The pharmacokinetic parameters for the investigation of the influence of food on bioavailability of OOD (AUC_(0–t)_ and C_max(0–t)_) and OTD (AUC_(0–12h)_ and C_max(0–12h)_) are listed in Table 2. The corresponding geometric mean values and 90% CIs are summarized in Table 5. The results show that for OOD the geometric mean ratio does not differ for the extent of bioavailability (AUC_(0–t)_) and is ~ 13% higher for C_max(0–t)_ under fed conditions in comparison to fasting conditions. The geometric mean ratios for OTD are ~ 29% and 34% higher for AUC_(0–12h)_ and C_max(0–12h)_ under fed conditions in comparison to fasting conditions, respectively. 

### Safety 

In all three studies, no serious or otherwise significant AEs or fatalities were reported, and no volunteers discontinued due to an AE. From the total 108 exposed subjects, 8 subjects experienced 9 AEs while on OOD, and 13 subjects experienced 13 AEs while on OTD. 

Study 1 (fasting conditions): With 4 observed AEs related to treatment in 3 subjects (1 case each of nausea, headache, vomiting, and itching; all classified as mild or moderate), both treatments were well tolerated when applied in fasted state. 

Study 2 (fed conditions): Minor safety issues with 11 treatment-related AEs in 11 subjects were observed. Those included headache (n = 7), itching (n = 3), and nausea (n = 1). One was considered moderate, the others as mild. 

Study 3 (multiple-dose administration): 7 AEs in 7 subjects were reported as treatment-related including headache (n = 5) and itching (n = 2). All were of mild intensity. 

## Discussion 

The bioavailability studies of OOD and OTD were designed and conducted according to current standards on investigation of bioequivalence and on pharmacokinetic evaluation of modified-release dosage forms [21, 22]. OOD (10 mg) was administered once (in the morning) and OTD (5 mg) twice (in the morning and evening) in order to reach the same total daily dose. Results show that the chosen methods, including the time points of blood sampling and the length of wash-out phase, were adequate to describe plasma profiles and bioavailability of the investigated products. 

The established oxycodone twice-daily (OTD) product used in the studies as the reference product is a single-unit matrix formulation where the release of oxycodone is biphasic, with an initial relatively fast release followed by a more controlled release, which determines the 12-hour dosing interval [7]. 

Compared to immediate-release formulations, the established twice-daily prolonged-release opioid products represent progress in terms of more stable plasma levels and more convenient dosing [13, 14]. At the same time, those products still bear disadvantages, such as end-of-dose failures and peak-to-trough fluctuations, that may be associated with breakthrough pain [23, 24]. Independently from these disadvantages, a large majority of patients in this therapeutic area prefer a simple and convenient once-daily dosing regimen [9, 25]. 

The new oxycodone once-daily (OOD) product is a multiple-unit pellet formulation. The oxycodone plasma concentration-time profile resulting from the new OOD shows the typical pattern of a once-daily formulation with a gradual increase during the first 4 – 6 hours followed by a plateau maintained for ~ 10 hours, and then a slow decline. OOD provides sustained drug concentrations over the dosing interval of 24 hours and avoids the initial fast release and the 12-hour trough levels associated with the twice-daily product, while showing a comparably slow decline after 16 hours. 

The absolute values for AUC were in the range of those reported for immediate- and controlled-release oxycodone [13, 14, 26, 27]. 

For the extent of bioavailability, the maximum plasma concentrations as well as for the concentrations at the end of the dosing interval, OOD could be classified as comparable to OTD considering the bioequivalence acceptance criteria of 80.00 – 125.00%. The study after multiple-dose administration indicates therapeutic dosage regimen conditions and shows the comparability of OOD and OTD. It could be assumed that OOD leads to comparable efficacy and safety and is therefore exchangeable when administered at the same total daily dose as OTD. This finding could be confirmed by results of a recent trial in patients [28]. The randomized, double-blind, cross-over trial in patients with malignant and nonmalignant moderate to severe chronic pain showed that OOD is at least equivalent to OTD regarding therapeutic efficacy and safety. 

Bioavailability of OOD was not affected after intake of a standard high-fat meal before administration. Extent of bioavailability and maximum plasma concentrations were comparable. After administration of OTD under fed conditions, higher values were observed for the extent of bioavailability (+29%) and for the maximum plasma concentrations (+34%) in comparison to the intake under fasting conditions. The relevancy of these differences could not finally be assessed even if the recommendation in the OTD’s summary of product characteristics [8] stated that tablets can be taken with or without food. 

Recently, HVD and PTF have become popular parameters [20] to describe the duration and fluctuation of prolonged-release characteristics of opioid formulations [29, 30]. Those two parameters have been proposed as indicators of decreased incidences of breakthrough pain and/or end-of-dose pain in clinical pain management, showing promising results for once-daily opioid formulations [30, 31]. Results show that OOD has a sufficiently long HVD and appropriate peak-trough values to indicate that stable and constant concentrations could be held over the relevant time period. 

Both tablet formulations were generally well tolerated. Incidence of AEs was lower with OOD. From the total 108 subjects exposed to OOD and OTD in the three studies, 21 subjects (19.4%) experienced any adverse event, thereof 8 subjects (7.4%) while under OOD and 13 subjects (12.0%) while under OTD. Reported adverse reactions were headache, itching, nausea, and vomiting, all transient and mostly of mild intensity. These are well-known side effects of opioids, especially when administered to opioid-naïve healthy volunteers. 

Whether long-acting opioids, such as oral prolonged-release preparations for once- or twice-daily dosing and transdermal systems, offer advantages over short-acting opioids in terms of efficacy and tolerability is still a matter of debate. Recent systematic review found no evidence supporting long-acting opioids’ superiority to short-acting ones in improving functional outcomes, reducing side effects or addiction [32]. However, prolonged-release formulations clearly have the benefit of less frequent dosing. As an easy, less frequent dosing schedule is preferred by chronic-pain patients [9, 25], longer-acting once-daily opioid preparations may nevertheless best fit patient requirements. 

In summary, it could be concluded that the developed OOD multiple-unit pellet prolonged-release tablet provides sustained and stable oxycodone plasma concentrations over 24 hours, being appropriate for simplified once-daily dosing regimen. The new OOD may add to physicians’ armamentarium of strong opioid products for patient-centered therapy in chronic pain with optimized dosing and better compliance. 

## Acknowledgment 

Medical writing support was provided by Stephan Döppenschmitt and Petra Roos and was funded by Develco Pharma Schweiz AG. 

## Conflict of interest 

MM, YG, and HR are employees of Develco Pharma Schweiz AG, the sponsor of the studies. BS, VG, and KS are employees of ACC GmbH, a contractor of Develco Pharma Schweiz AG. PK was the principal investigator, contracted by ACC GmbH. 

**Table 1.** Demographic data of subjects.

Parameter	Study 1 (fasting) N = 36	Study 2 (fed) N = 36	Study 3 (multiple dose) N = 36
Age (years)^a^	27.5 (18 – 54)	31.0 (20 – 54)	30.1 (19 – 49)
Gender (number of)			
Male	30	26	29
Female	6	10	7
Body mass index (kg)^a^	23.78 (19.5 – 27.4)	23.55 (19.5 – 27.4)	23.48 (19.4 – 27.6)
Weight (kg)^a^	76.3 (54 – 107)	71.4 (50 – 96)	72.2 (52 – 105)
Height (cm)^a^	178.8 (162 – 199)	173.9 (160 – 197)	175.1 (160 – 196)

^a^Arithmetic mean (range).

**Table 2.** Pharmacokinetic parameters of oxycodone after single-dose administration of OOD and OTD.

Parameter	Study 1 (fasting)		Study 2 (fed)	
	OOD	OTD	OOD	OTD
AUC_(0–t)_ (ng/mL×h)^a^	90.63 [95.78] (39.81 – 181.40)	76.47 [81.10] (39.08 – 197.35)	90.80 [95.64] (36.97 – 156.43)	88.36 [93.47] (37.17 – 158.45)
AUC_(0–∞)_ (ng/mL×h)^a^	99.85 [104.62] (48.06 – 193.83)	84.04 [88.18] (50.18 – 203.64)	98.51 [103.04] (45.43 – 161.93)	96.91 [101.50] (48.75 – 171.95)
Residual area (%)^b^	9.11 ± 4.689 (2.68 – 19.81)	8.90 ± 4.503 (2.38 – 22.12)	7.75 ± 3.642 (2.73 – 18.64)	8.66 ± 5.242 (3.02 – 23.76)
AUC_(0–12h)_ (ng/mL×h)^a^	NA	27.90 [29.05] (15.23 – 55.74)	NA	35.88 [37.33] (19.08 – 55.52)
C_max(0–t)_ (ng/mL)^a^	5.05 [5.23] (2.76 – 8.43)	4.92 [5.16] (2.61 – 9.97)	5.68 [5.89] (2.31 – 8.53)	5.65 [5.84] (2.94 – 9.68)
C_max(0–12h)_ (ng/mL)^a^	NA	3.87 [4.00] (2.22 – 7.77)	NA	5.20 [5.38] (2.94 – 7.97)
t_max(0–t)_ (h)^b^	9.6 ± 2.06 (4.0 – 12.0)	14.1 ± 3.00 (1.5 – 15.5)	9.1 ± 2.29 (7.0 – 14.0)	9.1 ± 6.52 (1.0 – 16.5)
t_max(0–12h)_ (h)^b^	NA	2.4 ± 1.04 (0.5 – 4.0)	NA	2.8 ± 1.39 (1.0 – 6.0)
T_1/2_ (h)^b^	7.1 ± 1.38 (4.5 – 10.3)	6.3 ± 1.39 (3.8 – 10.8)	6.5 ± 1.20 (4.3 – 9.3)	6.3 ± 1.76 (3.9 – 12.0)

^a^Geometric mean [arithmetic mean] (range); ^b^arithmetic mean ± standard deviation (range). OOD = oxycodone one daily; OTD = oxycodone twice daily; NA = not applicable.

**Table 3.** Pharmacokinetic parameters of oxycodone after multiple-dose administration of OOD and OTD.

Parameter	Study 3 (multiple dose)	
	OOD	OTD
AUC_ss(0–24h)_ (ng/mL×h)^a^	111.54 [116.22] (65.81 – 206.29)	98.05 [103.63] (54.45 – 228.06)
C_max,ss(0–24h)_ (ng/mL)^a^	6.76 [7.05] (4.43 – 13.73)	6.97 [7.42] (4.03 – 20.64)
C_τ,ss,24h_ (ng/mL)^a^	2.57 [2.77] (0.57 – 6.02)	2.39 [2.57] (1.29 – 5.96)
HVD_ss(0–24h)_ (h)^b^	18.9 ± 2.45 (11.0 – 23.2)	15.0 ± 3.37 (7.9 – 22.3)
PTF_ss(0–24h)_ (%) ^a^	95.60 [97.13] (71.43 – 168.85)	116.41 [117.76] (77.69 – 188.84)

^a^Geometric mean [arithmetic mean] (range); ^b^arithmetic mean ± standard deviation (range). OOD = oxycodone one daily; OTD = oxycodone twice daily.

**Table 4.** Comparison of bioavailability: Geometric mean ratios, 90% confidence intervals (CI), and residual coefficients of variation (CV_res_) of oxycodone after administration of OOD and OTD.

Study	Parameter	Geometric mean ratio (OOD/OTD)	90% CI	CV_res_ (%)
Study 1 (fasting)	AUC_(0–t)_	1.185	1.136 – 1.237	10.65
	C_max(0–t)_	1.025	0.962 – 1.093	16.12
Study 2 (fed)	AUC_(0–t)_	1.028	0.980 – 1.077	11.93
	C_max(0–t)_	1.007	0.965 – 1.051	10.71
Study 3 (multiple dose)	AUC_ss(0–24h)_	1.138	1.086 – 1.191	11.63
	C_max,ss(0–24h)_	0.970	0.919 – 1.023	13.43
	C_t,ss,24h_	1.078	0.977 – 1.190	25.17

OOD = oxycodone one daily; OTD = oxycodone twice daily.

**Table 5.** Influence of food: Geometric mean ratios, 90% confidence intervals (CI), and residual coefficients of variation (CV_res_) of oxycodone after administration of OOD or OTD under fasting or fed conditions.

Formulation	Parameter	Geometric mean ratio (fed/fasting)	90% CI	CV_res_ (%)
OOD	AUC_(0–t)_	1.002	0.878 – 1.144	34.64
	C_max(0–t)_	1.126	1.011 – 1.255	28.11
OTD	AUC_(0–12h)_	1.286	1.148 – 1.440	29.44
	C_max(0–12h)_	1.344	1.213 – 1.490	26.62

OOD = oxycodone one daily; OTD = oxycodone twice daily.
